# 

**Published:** 1848-10

**Authors:** 


### AMERICAN JOURNAL

OF

### Dental Science.

PUBLISHED UNDER THE AUSPICES OF THE

**American Society of Dental Surgeons.**

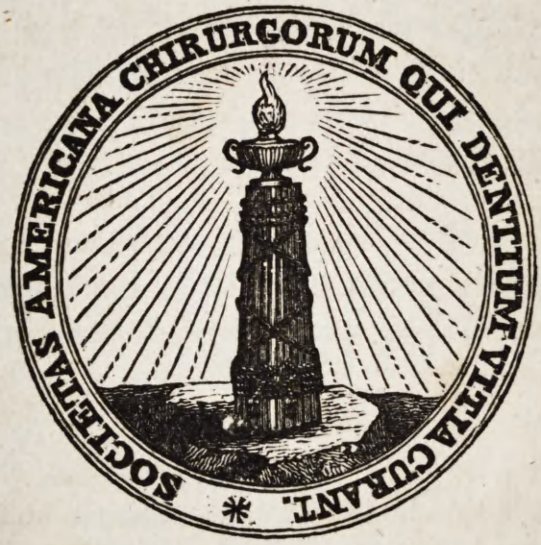

SURGEON GEN'L'S OFFICE
LIBRARY.
158314

VOLUME IX.—1848–9.

EDITORS:

CHAPIN A. HARRIS, M. D., D. D. S.
AMOS WESTCOTT, M. D., D. D. S.
WILLIAM H. DWINELLE, D. D. S.

BALTIMORE:
ARMSTRONG & BERRY,
PHILADELPHIA: LINDSAY & BLAKISTON.
JOHN W. WOODS, PRINTER.

W
1
AM 45 M

population of 40,000 to 50,000—the parents of the recent victims were carried off by the same disease, in '32–3. The observation, if correct, would seem to encourage the idea of the hereditary transmission of a susceptibility to this disease, and that its attacks are not indiscriminate, but measurably dependent upon constitutional diathesis.

*To our Readers.*—It is due to our subscribers to state the causes which have delayed the publication of the present number of the Journal to so late a period. Wishing to begin it with the proceedings of the last meeting of the American Society of Dental Surgeons, which had been taken down at length by a reporter employed for the purpose, we delayed putting any of the matter in the hands of the printer, five weeks beyond the time when it should have been commenced, and as the Baltimore Editor had not then received it, he wrote to the Recording Secretary, Dr. Westcott, for a copy of the minutes, which was promptly furnished. The matter was now put in the hands of the printer, who was then delayed three weeks for want of suitable paper. The printing of the second number of the present volume will be commenced immediately, and although that will necessarily be late in coming out, we hope to be able to furnish the future numbers to subscribers by the time they shall become due.

With regard to the report of the proceedings of the last meeting of the American Society of Dental Surgeons, we have not yet received it, and are wholly at a loss to imagine why it has been so long withheld.

*Cincinnati Agent.*—We would inform our friends and subscribers in the west and south-west, that Dr. J. M. Brown, corner of Fourth and Walnut streets, Cincinnati, Ohio, is authorized to receive subscriptions for this publication. In mentioning the name of Dr. Brown, we would remind our friends of the west that he has a large stock of materials, such as is used by the dentist, and we hope, should they have occasion to visit the Queen City, they will not fail to give him a call.

*Boston Agent.*—Mr. Joseph Burnett, Druggist, No. 33 Tremont Row, Boston, has consented to become an agent for the Journal, and is authorized to transact any business that pertains to it. We would also add that Mr. Burnett has the largest assortment of mineral teeth and dentists' materials of every description, to be found in New England, and we feel certain our friends in this part of the country will be glad of the opportunity, while ordering materials from Mr. B , to obtain information and transact any business which they may have with this publication.

*Travelling Agent.*—Mr. Henry P. Hardy, now on a tour through the southern and western states, is authorized to receive subscriptions for this periodical, and we hope that such of our subscribers as may be in arrears, and upon whom he may call, will avail themselves of the opportunity to pay the amount of their indebtedness, and their subscription to the present volume. At any rate, we are sure that it is only necessary to say to them that the money is needed for the publication, to induce them to do it.

### WORKS ON DENTAL SURGERY,

### RECENTLY PUBLISHED,

### BY LINDSAY & BLAKISTON,

PHILADELPHIA.

### HARRIS' PRINCIPLES AND PRACTICE

OF

### DENTAL SURGERY,

**Third Edition, Revised, Improved and Greatly Enlarged.**

ILLUSTRATED BY

**156 WELL EXECUTED WOOD ENGRAVINGS,**

**In One Volume, Octavo.**

The present edition of the above work, enlarged and improved, as it has been, by the author, may be safely pronounced the most comprehensive, and at the same time the most concise treatise on the subject of Dental Surgery which has yet appeared in the English language. It embodies not only the sound views of the author, founded upon well directed observations during a constant practice of many years, but those also of a great number of writers of eminence, freed from errors which have too often misled students, and laid the foundation of a mischievous practice. The necessity for a treatise from the pen of any intelligent member of the profession of Dental Surgery, in all its branches, has been long wanted, and this work will be hailed with pleasure and read with profit by every one interested in the subject. A glance at the plan of the work, and its contents, will serve to show the wide field traversed by the author. This treatise will not only meet with a ready and extensive sale, but will become a standard work, for it furnishes the basis of a sound and thorough practice.—*Saturday Courier.*

### THE SURGICAL AND MECHANICAL TREATMENT

OF

### THE TEETH:

**INCLUDING DENTAL MECHANICS.**

**With one hundred and thirty-nine Illustrations.**

**BY JAMES ROBINSON,**

Surgeon Dentist to the Metropolitan Hospital, &c. &c.

### ARTHUR'S MANUAL

### OF THE DISEASES OF THE TEETH,

Including a Description of their Structure, Modes of Treatment, &c. &c.

A small Pocket Volume.

"It gives us pleasure to state that we have seldom read a popular treatise on the Teeth, that contains so much useful and valuable information as does this. It is a work which every family should have, and we can recommend it too, to the members of the dental profession, as well worthy of perusal."—*Dental Intelligencer.*

"It gives us pleasure to announce the publication of this work, which we regard, so far as we have had an opportunity of examining it, as one of the best popular treatises on the subject extant. As a literary production, it is highly creditable to its author."—*Amer. Jour. of Dental Science.*

